# Organocatalytic Upgrading of Furfural and 5-Hydroxymethyl Furfural to C_10_ and C_12_ Furoins with Quantitative Yield and Atom-Efficiency

**DOI:** 10.3390/ijms16047143

**Published:** 2015-03-30

**Authors:** Hongjun Zang, Eugene Y. X. Chen

**Affiliations:** Department of Chemistry, Colorado State University, Fort Collins, CO 80523-1872, USA; E-Mail: zanghongjun@tjpu.edu.cn

**Keywords:** furfural, 5-hydroxymethyl furfural (HMF), umpolung coupling, organocatalysis, *N*-heterocyclic carbene, ionic liquid, biomass, biorefining

## Abstract

There is increasing interest in the upgrading of C_5_ furfural (FF) and C_6_ 5-hydroxymethyl furfural (HMF) into C_10_ and C_12_ furoins as higher energy-density intermediates for renewable chemicals, materials, and biofuels. This work utilizes the organocatalytic approach, using the *in situ* generated *N*,*S*-heterocyclic carbene catalyst derived from thiazolium ionic liquids (ILs), to achieve highly efficient self-coupling reactions of FF and HMF. Specifically, variations of the thiazolium IL structure have led to the most active and efficient catalyst system of the current series, which is derived from a new thiazolium IL carrying the electron-donating acetate group at the 5-ring position. For FF coupling by this IL (0.1 mol %, 60 °C, 1 h), when combined with Et_3_N, furoin was obtained in >99% yield. A 97% yield of the C_12_ furoin was also achieved from the HMF coupling by this catalyst system (10 mol % loading, 120 °C, 3 h). On the other hand, the thiazolium IL bearing the electron-withdrawing group at the 5-ring position is the least active and efficient catalyst. The mechanistic aspects of the coupling reaction by the thiazolium catalyst system have also been examined and a mechanism has been proposed.

## 1. Introduction

Biofuran aldehydes (or furaldehydes), particularly furfural (FF) and 5-hydroxymethyl furfural (HMF) [1,2,3,4,5,6,7,8,9,10,11], which are primarily derived from dehydration of C_5_ and C_6_ (poly)sugars, have emerged as two key renewable biomass building blocks for biorefining towards addressing global challenges to develop technologically and economically feasible routes for converting nonfood lignocellulosic biomass into feedstock chemicals, sustainable materials, and liquid fuels [12,13,14,15,16,17]. However, such C_5_ and C_6_ furaldehydes lead to, upon upgrading, relatively low carbon-number and low energy-density bio-products or fuels. Hence, there is increasing interest in upgrading of FF and HMF into higher molecular weight and higher energy-density kerosene/jet (C_8_ to C_16_) or diesel (up to C_22_) intermediates or fuels. This upgrading can be accomplished by chain extension involving new C–C bond formation through coupling with other organic compounds [18,19,20,21]. Considering the fact that such furaldehydes cannot undergo self-aldol condensation due to lack of α-hydrogen, Dumesic and co-workers utilized cross-aldol condensation of HMF with enolizable organic compounds such as acetone in the presence of an alkaline catalyst, followed by hydrodeoxygenation (HDO) processes, to upgrade HMF into C_9_ to C_15_ liquid alkane fuels [22]. Alternatively, opening the furan rings first under mild conditions, followed by HDO, produced alkanes more selectively [23]. Direct (reductive) coupling of two FF molecules by metal catalysts has also been utilized, leading to formation of Pinacol coupling products as a mixture of C_10_ alcohols, which can be subsequently converted into C_8–14_ linear and branched alkanes via the HDO process [24].

**Scheme 1:**
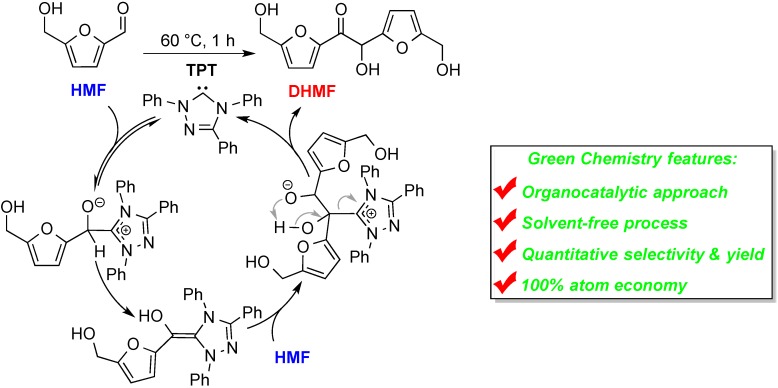
Solvent-free NHC-catalyzed self-condensation of HMF to DHMF and depicted umpolung coupling catalytic cycle.

Departing from the widely employed metal-based catalyst systems, the application of organocatalysis in biomass conversion and upgrading into sustainable chemicals, materials, and biofuels has come to light recently [25]. The organocatalytic approach employs small-molecular organic compounds as efficient metal-free “greener” catalysts to promote catalytic chemical transformations [26,27,28,29,30,31,32], showing potentials of using relatively non- or less toxic, more environmentally benign, atom-economical and more sustainable organocatalysis for biorefining [25,33,34,35]. For instance, we recently found that a room-temperature ionic liquid (IL), 1-ethyl-3-methylimidazolium acetate ([EMIM]OAc), can readily couple (or dimerize) two HMF molecules together to form C_12_ 5,5'-dihydroxymethyl furoin (DHMF) in high conversion and selectivity [36]. The true catalyst for this coupling reaction was uncovered to be the *N*-heterocyclic carbene (NHC), 1-ethyl-3-methylimidazol-2-ylidene, which exists in an equilibrium with [EMIM]OAc and is stabilized by HOAc. Understanding of the coupling mechanism and catalytically active specie responsible for the [EMIM]OAc-promoted HMF self-coupling led to a much more effective HMF upgrading process; thus, use of a discrete, stable NHC, 1,3,4-triphenyl-4,5-dihydro-1H-1,2,4-triazol-5-ylidene (TPT) [37,38], afforded DHMF with a quantitative selectivity and nearly quantitative isolated yield, with 1 mol % of TPT in a solvent-free process at 60 °C for 1 h [33]. The mechanism of this NHC-catalyzed direct coupling of HMF is outlined in Scheme 1, which resembles that for the NHC-catalyzed benzoin condensation reaction (*vide infra*). Significantly, as a higher energy-density C_12_ fuel intermediate, DHMF has been converted to oxygenated diesels by hydrogenation, etherification and esterification, as well as to high-quality alkane fuels; the latter transformation was achieved by the HDO process in water catalyzed by Pt/C and TaOPO_4_, which converted DHMF to linear hydrocarbons, with 96% selectivity to C_10–12_ linear alkanes: 27.0% *n*-decane, 22.9% *n*-undecane, and 45.6% *n*-dodecane [33].

**Scheme 2:**
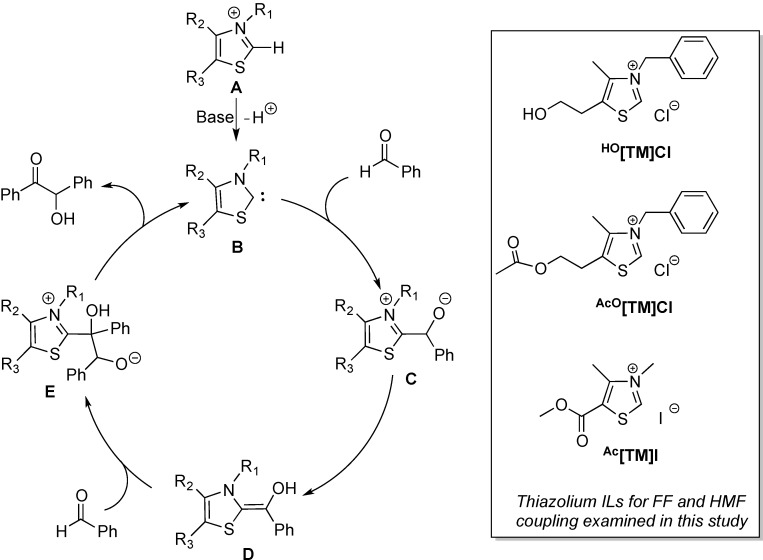
Outlined catalytic cycle of the benzoin condensation through the Breslow intermediate (**D**) and the three thiazolium ILs investigated in this study as the precatalysts for coupling of furaldehydes.

ILs exhibit the unique ability to dissolve lignocellulosic biomass [39,40] and related carbohydrates [41,42] under relatively mild conditions; this feature, plus several other concurrent advantages (e.g., as designable and recyclable solvents with low volatility and toxicity), has attracted much interest [43,44,45], particularly in the pursuit of renewable energy and sustainable chemicals from plant biomass [46,47]. Pertinent to the organocatalytic upgrading of furaldehydes, ILs are also precursors to the NHC catalysts that can efficiently promote benzoin condensation, a direct coupling reaction between two aldehydes [26,27,28,29,30,31,32,48]. Scheme 2 outlines the mechanism for the NHC-catalyzed benzoin condensation of benzaldehyde originally proposed by Breslow [49,50,51]. In this mechanism, thiazolium salt **A** is deprotonated by a strong base (e.g., Et_3_N) to form thiazolin-2-ylidene NHC catalyst **B**. Nucleophilic attack of the aldehyde by **B** generates the carbene-aldehyde zwitterionic adduct **C**, followed by subsequent protonation/deprotonation or proton transfer, which affords the Breslow intermediate, **D**. This amino enol intermediate functions as an acyl anion equivalent and attacks a second aldehyde, forming zwitterionic adduct **E** [49,50,51]. After the proton transfer and the elimination of the benzoin product, the NHC catalyst **B** is regenerated. This process is also termed aldehyde umpolung (polarity inversion) that converts the electrophilic carbonyl carbon to a nucleophilic center as an acyl anion equivalent. As for the biomass-derived furaldehydes, benzoin condensation of FF is catalyzed by NHCs in a similar fashion [52,53,54]. For example, the methanol adduct of TPT, TPT(MeOH), was used to catalyze condensation of FF to give furoin in 78% yield [55]. On the other hand, 3-benzyl-5-(2-hydroxyethyl)-4-methylthiazolium bromide, ^HO^[TM]Br, in combination with Et_3_N, was employed for benzoin condensation of FF, achieving 90% yield of furoin [56].

To explore inexpensive alternatives to the discrete NHC catalysts such as TPT for furaldehyde umpolung coupling, we arrived at thiazolium IL ^HO^[TM]Cl, an analog of thiamine (Vitamin B1). The central objective of this work was to investigate activity and selectivity of the structurally modified ^HO^[TM]Cl derivatives, ^AcO^[TM]Cl bearing an electron-donating group at the 5-ring position and ^Ac^[TM]I carrying an electron-withdrawing group at the 5-ring position (Scheme 2), in the self-coupling of FF and HMF. We reasoned that, by tuning the electronic feature of the thiazolium ring, the nucleophilicity of the corresponding NHC catalyst derived from deprotonation could be modulated, thus providing an opportunity to discover more activity and/or selective catalysts for coupling the biomass-derived furaldehydes.

## 2. Results and Discussion

### 2.1. Synthesis of Two New Thiazolium ILs ^AcO^[TM]Cl and ^Ac^[TM]I 

**Scheme 3:**
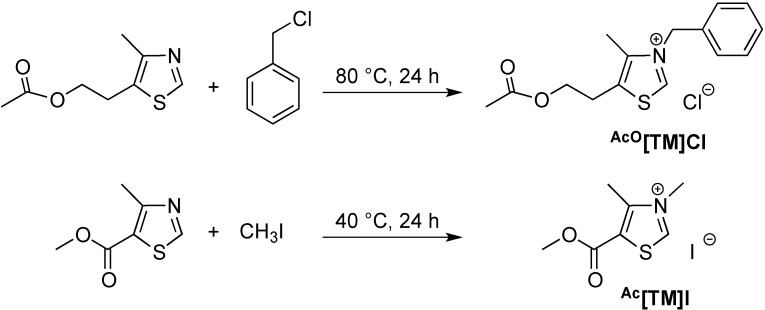
Synthesis of two new thiazolium salts as the precatalysts for coupling of furaldehydes.

**Figure 1:**
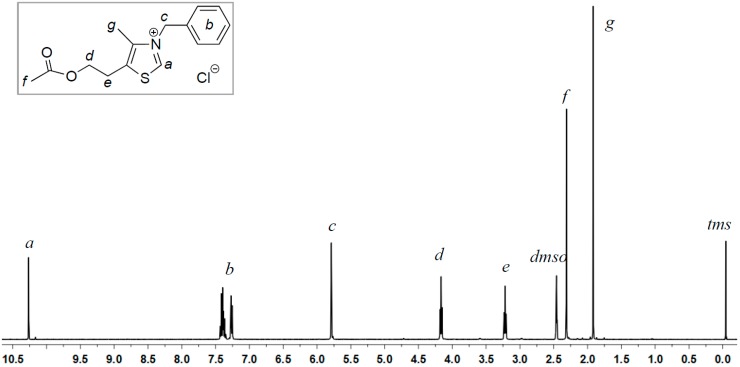
^1^H NMR (DMSO-*d*_6_) of ^AcO^[TM]Cl.

**Figure 2:**
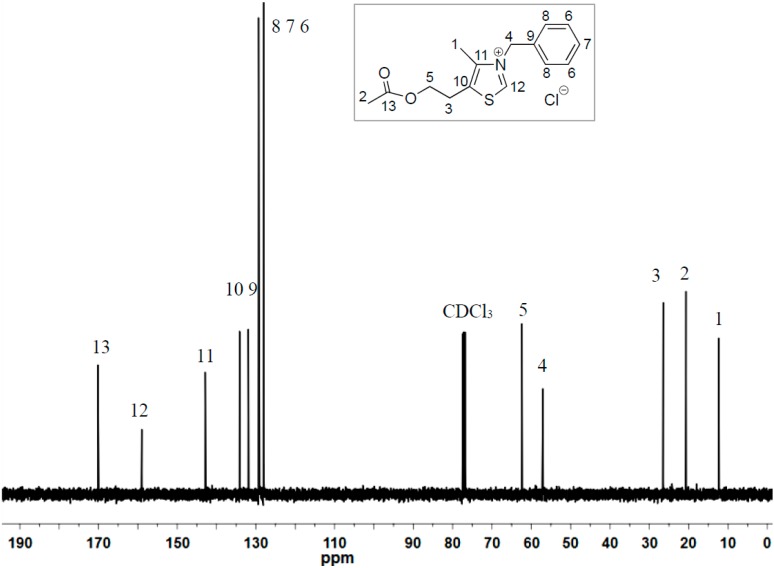
^13^C NMR (CDCl_3_) of ^AcO^[TM]Cl.

The parent thiazolium salt ^HO^[TM]Cl carries the 2-hydroxyethyl group at the 5-position of the methylthiazolium ring. To examine if the protic OH group facilitates or hampers the coupling reaction of furaldehydes, we designed the synthesis of its acetate derivative ^AcO^[TM]Cl. Alkylation of the corresponding substituted methylthiazole with benzyl chloride produced ^AcO^[TM]Cl in 81% isolated yield (Scheme 3). ^1^H NMR (DMSO-*d*_6_, Figure 1) and ^13^C NMR (DMSO-*d*_6_, Figure 2) spectra confirm the formation of the clean, desired product, with all the resonances assigned to the expected protons and carbons, respectively (see Experimental Section). Noteworthy is the chemical shift of the C2-H (*i.e.*, NC*H*S) at 10.26 ppm, indicative of the acidic nature of this proton.

**Figure 3:**
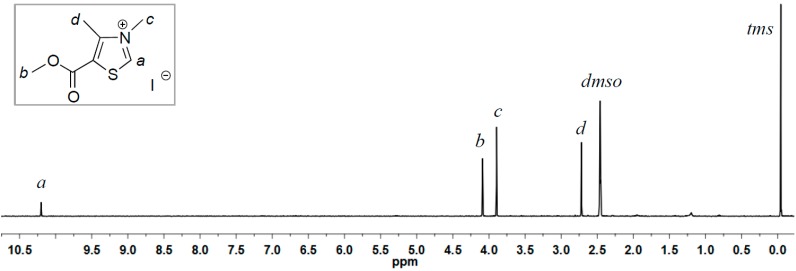
^1^H NMR (DMSO-*d*_6_) of ^Ac^[TM]I.

**Figure 4:**
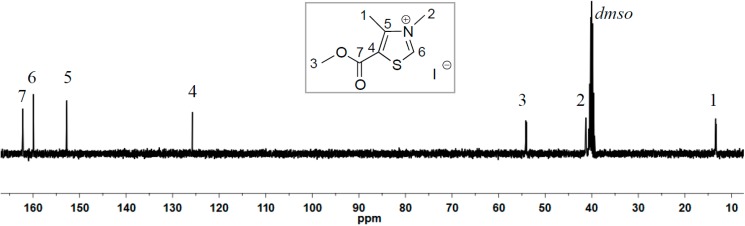
^13^C NMR (DMSO-*d*_6_) of ^Ac^[TM]I.

As both ^HO^[TM]Cl and ^AcO^[TM]Cl carry an electron-donating group at the 5-ring position, we designed the synthesis of ^Ac^[TM]I with the electron-withdrawing methoxycarbonyl (acetyl type) group attached to the 5-ring position (Scheme 3). Initial attempts to alkylate the corresponding methylthiazole with benzyl chloride failed to produce the anticipated thiazolium chloride salt, as a result of the electron-deficient nature of the thiazole ring with the methoxycarbonyl attached to it. In turn, we employed the stronger alkylating reagent, MeI, successfully producing the corresponding thiazolium iodide salt, ^Ac^[TM]I. As can be seen from ^1^H NMR (DMSO-*d*_6_, Figure 3) and ^13^C NMR (DMSO-*d*_6_, Figure 4) spectra, the formation of the clean, desired product is achieved. As anticipated, the acidic C2-H (*i.e.*, NC*H*S) is largely downfield-shifted to 10.02 ppm, consistent with the chemical shift of the same proton observed for ^HO^[TM]Cl and ^AcO^[TM]Cl.

### 2.2. Coupling Reaction of FF to Furoin by ^HO^[TM]Cl, ^AcO^[TM]Cl, and ^Ac^[TM]I

The self-coupling of FF to 1,2-Di(furan-2-yl)-2-hydroxyethanone (furoin) by the thiazolium salt in combination with a base was carried out under neat conditions (no solvent), the results of which were summarized in Table 1. The acetate derivative, ^AcO^[TM]Cl, when combined with 2 equiv of Et_3_N, was found to be highly effective to catalyze the FF-to-furoin coupling reaction. Specifically, with a loading of 1 mol % pre-catalyst, furoin was achieved in 99% yield (by NMR) at 80 °C in 3 h (entry 1, Table 1). Lowering the pre-catalyst loading to 0.5 and 0.1 mol % while keeping the temperature and time the same still afforded furoin in quantitative yield (entries 2 and 3, Table 1). When carried out at 60 °C for 1 h, the furion yield with a low pre-catalyst loading of 0.1 mol % depended on the amount of the base added, varying from 81% (entry 4), 86% (entry 5), to >99% (entry 6) when the amount of Et_3_N was increased from 1, 2, and 4 equivalents (relative to the pre-catalyst), respectively. Other bases such as KO^t^Bu and 1,8-diazabicyclo[5.4.0]undec-7-ene (DBU) were also examined but found to be much inferior to Et_3_N (entries 7 and 8 *vs.* entry 5, Table 1).

**Table 1:** Results of self-coupling of FF to furoin catalyzed by thiazolium ILs and base.

Entry	Pre-Catalyst (mol %)	Base (mol %)	Temperature (°C)	Time (h)	Furoin Yield (%)
1	^AcO^[TM]Cl, 1	Et_3_N, 2	80	3	99
2	^AcO^[TM]Cl, 0.5	Et_3_N, 1	80	3	99
3	^AcO^[TM]Cl, 0.1	Et_3_N, 0.2	80	3	>99
4	^AcO^[TM]Cl, 0.1	Et_3_N, 0.1	60	1	81
5	^AcO^[TM]Cl, 0.1	Et_3_N, 0.2	60	1	86
6	^AcO^[TM]Cl, 0.1	Et_3_N, 0.4	60	1	>99
7	^AcO^[TM]Cl, 0.1	KO^t^Bu, 0.2	60	1	60
8	^AcO^[TM]Cl, 0.1	DBU, 0.2	60	1	56
9	^HO^[TM]Cl, 0.5	Et_3_N, 1	80	3	99
10	^HO^[TM]Cl, 0.1	Et_3_N, 0.4	60	1	84
11	^Ac^[TM]I, 0.1	Et_3_N, 0.2	80	3	0
12	^Ac^[TM]I, 0.5	Et_3_N, 1	80	3	16
13	^Ac^[TM]I, 5	Et_3_N, 10	80	3	49
14	^Ac^[TM]I, 10	Et_3_N, 20	80	3	84

The performance of ^AcO^[TM]Cl was compared with the benchmark thiazolium IL ^HO^[TM]Cl. Under relatively high pre-catalyst loading conditions (0.5 mol % or above) at 80 °C for 3 h, the two catalyst systems were indistinguishable, with both achieving quantitative furoin yields under the same conditions (entry 9 *vs.* 2, Table 1). However, monitoring the reaction by ^1^H NMR showed the coupling reaction by the catalyst derived from ^AcO^[TM]Cl is much faster and efficient than the coupling by ^HO^[TM]Cl. Thus, with 0.5 mol % of the pre-catalyst loading in combination with 1 mol % of Et_3_N at 80 °C, the coupling reaction by ^HO^[TM]Cl achieved only 18% furoin yield after 20 min, corresponding to a turn-over frequency (TOF) of 108 h^−1^, while the coupling by ^AcO^[TM]Cl afforded furoin in 83% yield over the same time period of 20 min, with TOF = 498 h^−1^, thus being 4.6 times faster. Furthermore, employing the best-performing conditions found for ^AcO^[TM]Cl (0.1 mol % pre-catalyst, 0.4 mol % Et_3_N, 60 °C, 1 h), the ^HO^[TM]Cl-based catalyst system produced furoin in 84% yield (entry 10, Table 1), as compared to >99% yield achieved by ^AcO^[TM]Cl (entry 6, Table 1).

The thiazolium derivative carrying an electron-withdrawing group on the ring, ^Ac^[TM]I, was much inferior to either ^HO^[TM]Cl or ^AcO^[TM]Cl. For instance, with a low pre-catalyst loading of 0.1 mol %, the ^Ac^[TM]I-based system was ineffective for the coupling reaction (entry 11, Table 1). Increasing the pre-catalyst loading to 0.5, 5, and 10 mol %, the furoin yield was enhanced gradually to 16, 49, and 84%, respectively (entries 12, 13, and 14, Table 1). This large reduction in the catalytic performance by ^Ac^[TM]I could be attributable to the electron-withdrawing substitute that renders low nucleophilicity of the corresponding NHC catalyst. Overall, for the current three thiazolium ILs investigated for the FF coupling reaction, the catalytic performance follows this order: ^AcO^[TM]Cl > ^HO^[TM]Cl >> ^Ac^[TM]I.

### 2.3. Coupling Reaction of HMF to DHMF by ^HO^[TM]Cl, ^AcO^[TM]Cl, and ^Ac^[TM]I

The self-coupling of HMF to DHMF by the thiazolium salt in combination with a base was also carried out under neat conditions (no solvent), the results of which are summarized in Table 2. The coupling of HMF to DHMF is a much more challenging reaction than the self-coupling of FF, requiring the catalyst in higher loadings. Specifically, with 1 and 5 mol % loadings of ^AcO^[TM]Cl in combination with 2 equivalents of Et_3_N at 80 °C for 3 h, DHMF was achieved in only 34% and 50% yield, respectively (entries 1 and 2, Table 2). Increasing the catalyst loading to 10 mol % significantly enhanced the DHMF yield to 94% (entry 3, Table 2). Increasing the reaction temperature to 100 and 120 °C improved the DHMF yield somewhat to 96% and 97%, respectively (entries 4 and 5, Table 2). Again, other bases such as KO^t^Bu and DBU gave inferior catalytic performances relative to Et_3_N (entries 6, 7, and 8 *vs.* entries 3 and 4, Table 2).

**Table 2:** Results of self-coupling of HMF to DHMF catalyzed by thiazolium ILs and base.

Entry	Pre-Catalyst (mol %)	Base (mol %)	Temperature (°C)	Time (h)	Furoin Yield (%)
1	^AcO^[TM]Cl, 1	Et_3_N, 2	80	3	34
2	^AcO^[TM]Cl, 5	Et_3_N,10	80	3	50
3	^AcO^[TM]Cl, 10	Et_3_N, 20	80	3	94
4	^AcO^[TM]Cl, 10	Et_3_N, 20	100	3	96
5	^AcO^[TM]Cl, 10	Et_3_N, 20	120	3	97
6	^AcO^[TM]Cl, 10	KO^t^Bu, 20	80	3	89
7	^AcO^[TM]Cl, 10	KO^t^Bu, 20	100	3	93
8	^AcO^[TM]Cl, 10	DBU, 20	80	3	86
9	^HO^[TM]Cl, 10	Et_3_N, 20	80	3	93
10	^HO^[TM]Cl, 10	Et_3_N, 20	120	3	97
11	^Ac^[TM]I, 0.5	Et_3_N, 1	80	3	37
12	^Ac^[TM]I, 10	Et_3_N, 20	80	3	86
13	^Ac^[TM]I, 10	KO^t^Bu, 20	80	3	59
14	^Ac^[TM]I, 10	DBU, 20	80	3	71

For the HMF coupling reaction, ^HO^[TM]Cl performed comparably to ^AcO^[TM]Cl, achieving essentially the same DHMF yields under identical conditions (entry 9 *vs.* 3, entry 10 *vs.* 5, Table 2). On the other hand, based on the coupling results (entries 11–14, Table 2), ^Ac^[TM]I was again much inferior to either ^HO^[TM]Cl or ^AcO^[TM]Cl. Overall, for the current three thiazolium ILs investigated for the HMF coupling reaction, the catalytic performance follows this order: ^AcO^[TM]Cl ~ ^HO^[TM]Cl >> ^Ac^[TM]I.

### 2.4. Proposed Mechanism for FF and HMF Coupling Reaction

**Figure 5:**
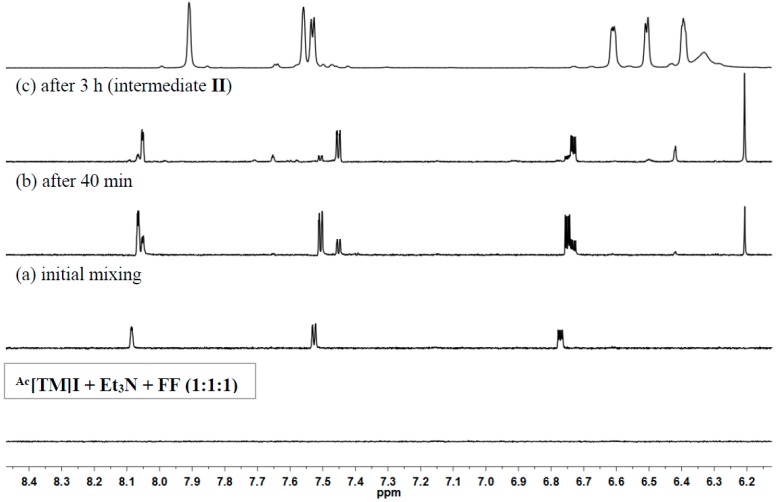
Overlay of ^1^H NMR (DMSO-*d*_6_) spectra in the furan and methide proton region of the reaction of ^Ac^[TM]I + Et_3_N + FF (1:1:1) at 80 °C leading to formation of intermediate **II** and furoin (upon addition second equivalent of FF).

**Scheme 4:**
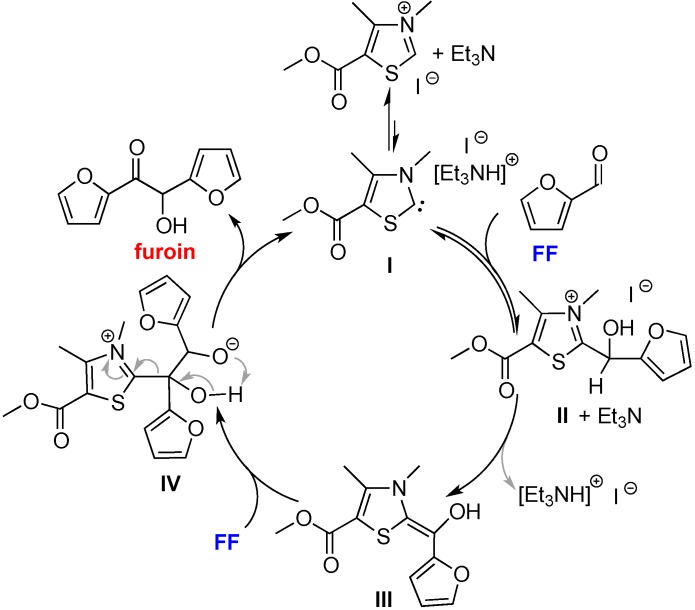
Proposed mechanism for coupling of FF by ^Ac^[TM]I/Et_3_N.

To provide further insight into the mechanism of the coupling reaction of FF by the thiazolium/Et_3_N system, we monitored the reaction of ^Ac^[TM]I + Et_3_N + FUR (1:1:1) at 80 °C by ^1^H NMR (Figure 5). The ^1^H NMR spectrum of the initial mixture showed a mixture of starting reagents (Figure 5a). As the reaction progresses, formation of a new species (from spectrum (a) to (b) to (c)) became apparent; the NMR characteristics of this species (δ 8.05 (dd, 1H), 7.45 (dd, 1H), 6.73 (dd, 1H) ppm for the three furan ring protons, δ 6.21 (s, 1H) for the methide proton (C*H*OH), δ 3.48 (s, 3H) for the methoxy group (OC*H_3_*), δ 2.88 (s, 3H) for the methyl group attached to *N* (NC*H_3_*), and δ 2.28 (s, 3H) for the methyl group on the ring (C=C–C*H_3_*).) are consistent with the NHC–FF adduct, intermediate **II** (Scheme 4). Addition of a second equivalent of FF to the resulting intermediate led to immediate formation of the final coupling product, furoin (Figure 5d).

The above results and the identification of intermediate **II** led to the coupling mechanism proposed in Scheme 4, which is consistent with the mechanism proposed for the self-coupling of HMF by [EMIM]OAc [36]. Specifically, the NHC catalyst (**I**) is generated by *in situ* deprotonation of ^Ac^[TM]I with Et_3_N. Nucleophilic addition of the carbene **I** to the carbonyl group of FF generates a zwitterionic tetrahedral intermediate, which is protonated by [Et_3_NH]^+^ to intermediate **II** and regeneration of Et_3_N. Next, intermediate **II** is deprotonated by Et_3_N to form a nucleophilic enol (**III**), the Breslow intermediate. This enol is an acyl anion equivalent (nucleophilic), thus attacking the carbonyl group of a second FF molecule to form another tetrahedral intermediate (**IV**). Collapse of this tetrahedral intermediate, via proton transfer and elimination of **I**, produces furoin and regenerates the NHC catalyst, thus closing the catalytic cycle (Scheme 4). The observation of intermediate **II** from the 1:1:1 reaction of ^Ac^[TM]I + Et_3_N + FF and immediate formation of furoin, without observing the intermediate **IV** upon addition of the second equivalent of FF, suggested that the steps from **I** to **II** are fast (and reversible), relative to the second half of the catalytic cycle involving addition of the second FF molecule.

## 3. Experimental Section

### 3.1. Materials, Reagents, and Methods

All syntheses and manipulations of air- and moisture-sensitive materials were carried out in flamed Schlenk-type glassware on a dual-manifold Schlenk line, on a high-vacuum line, or in an inert gas (Ar or N_2_)-filled glovebox. NMR-scale reactions were conducted in Teflon-valve-sealed J. Young-type NMR tubes. HPLC-grade organic solvents were first sparged extensively with nitrogen during filling 20 L solvent reservoirs and then dried by passage through activated alumina (for Et_2_O, THF, and CH_2_Cl_2_) followed by passage through Q-5 supported copper catalyst (for toluene and hexanes) stainless steel columns. DMSO-*d*_6_ was first degassed and dried over CaH_2_, followed by vacuum distillation (CaH_2_ was filtered off before the distillation). NMR spectra were recorded on a Varian Inova 300 (FT 300 MHz, ^1^H; 75 MHz, ^13^C) or a Varian Inova 400 MHz spectrometer. Chemical shifts for ^1^H and ^13^C spectra were referenced to internal NMR solvent residual resonances and reported as parts per million relative to SiMe_4_ (TMS). High-resolution mass spectrometry (HRMS) data were collected on an Agilent 6220 Accurate time-of-flight LC/MS spectrometer.

Furfural (FF, Alfa Aesar, Ward Hill, MA, USA), 5-hydroxymethyl furfural (HMF, Acros Organics, Waltham, MA, USA), Et_3_N (Acros Organics), KO*^t^*Bu (Acros Organics), 1,8-diazabicyclo [5.4.0] undec-7-ene (DBU, Acros Organics), methyl iodide (Acros Organics), 5-(2-acetoxyethyl)-4-methylthiazole (TCI America, Montgomeryville, PA, USA), 3-benzyl-5-(2-hydroxyethyl)-4-methylthiazolium chloride (^HO^[TM]Cl, Alfa Aesar), benzyl chloride (Acros Organics), and 4-methylthiazole-5-carboxylate (Oxchem Corp, Irwindale, CA, USA) were purchased from the respective commercial vendors and used as received.

### 3.2. Synthesis of Ionic Liquid ^AcO^[TM]Cl

5-(2-Acetoxyethyl)-4-methylthiazole (1.85 g, 10.0 mmol) was mixed with an excess of benzyl chloride (6.33 g, 50.0 mmol) in a round-bottom flask. The mixture was heated at 80 °C for 24 h, during which time precipitates formed. The solid was filtered off and washed with ethyl acetate. After drying under vacuum, ^AcO^[TM]Cl was obtained as a white solid; yield: 2.51 g (81%).

^1^H NMR (DMSO-*d*_6_): δ 10.26 (s, 1H, thiazole ring proton), 7.25–7.45 (m, 5H, *Ph*), 5.77 (s, 2H, C*H_2_*Ph), 4.12 (t, *J* = 4.5 Hz, 2H, OC*H_2_*), 3.22 (t, *J* = 4.5 Hz, 2H, OCH_2_C*H_2_*), 2.31 (s, 3H, C*H_3_*C=O), 1.92 (s, 3H, C=C–*CH_3_*). ^13^C NMR (DMSO-*d*_6_): δ 169.9 (*C*=O), 158.6 (S*C*N), 142.4 (N*C*=C), 133.8 (S*C*=C), 131.1, 129.3, 129.1, 128.1 (Ph), 62.4 (O*C*H_2_), 57.0 (*C*H_2_Ph), 26.2 (OCH_2_*C*H_2_), 20.6 (*C*H_3_C=O), 12.4 (C=C–*C*H_3_). HRMS calculated for C_15_H_18_ClNO_2_S [M-H]^−^: 310.0674; found: 310.0670.

### 3.3. Synthesis of Ionic Liquid ^Ac^[TM]I

4-Methylthiazole-5-carboxylate (1.0 g, 32 mmol) was mixed with an excess of methyl iodide (2.0 mL) in a round-bottom flask, and the mixture was heated at 40 °C for 24 h. After removal of the excess methyl iodide, the partly crystalline residue was dissolved in absolute alcohol. On careful addition of diethyl ether, the product crystallized in large light yellow needles (0.85 g, 45% yield on the first crop).

^1^H NMR (DMSO-*d_6_*): δ 10.02 (s, 1H, thiazole ring-H), 4.09 (s, 1H, OC*H_3_*), 3.89 (s, 3H, NC*H_3_*), 2.75 (s, 3H, C=C–C*H_3_*). ^13^C NMR (DMSO-*d*_6_): δ 162.3 (*C*=O), 159.9 (S*C*N), 152.9 (N*C*=C), 125.6 (S*C*=C), 54.2 (O–*C*H_3_), 41.3 (N*C*H*_3_*), 13.1 (C=C–*C*H_3_). HRMS calculated for C_7_H_10_INO_2_S [M + H]^+^: 299.9477; found: 299.8741.

### 3.4. Typical Procedure for Coupling Reaction of FF into Furoin

The coupling reaction was carried out under solvent-free (neat) conditions. In a typical coupling experiment, FF (0.77 g, 8.0 mmol), ^AcO^[TM]Cl (25 mg, 0.08 mmol), and Et_3_N (2.23 μL, 0.16 mmol) were loaded into a 20 mL vial in the glovebox. The vial was sealed, taken out of the glovebox, and then put in a temperature-controlled orbit shaker. The reaction mixture was shaken (300 rpm) at 80 °C for 3 h. After the reaction, the solidified product was washed with 10 mL hexanes and filtered. After drying in vacuum, furoin was obtained in 99% yield as a bright-yellow powder. ^1^H NMR for furoin (CDCl_3_) [33,55]: δ 7.62, 7.38, 7.26, 6.54, 6.41, 6.36 (dd, 6H, furan ring H), 5.80 (s, 1H, C*H*OH), 4.19 (br s, 1H, CHO*H*). ^1^H NMR for furoin (DMSO-*d*_6_): δ 8.00 (d, 1H), 7.60 (d, 1H), 7.53 (d, 1H), 6.79 (d, 1H), 6.42 (d, 2H), 6.12 (d, *J* = 6.2 Hz, 1H, CHO*H*), 5.79 (d, *J* = 6.3 Hz, 1H, C*H*OH).

### 3.5. Typical Procedure for Coupling Reaction of HMF into DHMF

The coupling reaction was carried out under solvent-free (neat) conditions. In a typical coupling experiment, HMF (0.126 g, 1.00 mmol), ^AcO^[TM]Cl (0.0311 g, 0.1 mmol), and Et_3_N (27.4 μL, 0.2 mmol) were loaded into a 20 mL vial in the glovebox. The vial was sealed, taken out of the glovebox, and then put in a temperature-controlled orbit shaker. The reaction mixture was shaken (300 rpm) at 80 °C for 3 h. After the reaction, the solidified product was washed with 10 mL toluene to remove the residual catalyst and any unreacted HMF. DHMF (94% yield) was obtained as white powder after filtration and vacuum drying. ^1^H NMR for DHMF (DMSO-*d*_6_) [36]: δ 7.50, 6.50, 6.34, 6.22 (d, 4H, furan ring H), 6.06 (d, *J* = 6.2 Hz, 1H, CHO*H*), 5.73 (d, *J* = 6.2 Hz, 1H, C*H*OH), 5.50 (t, *J* = 5.9 Hz, 1H, CH_2_O*H*), 5.18 (t, *J* = 5.7 Hz, 1H, CH_2_O*H*), 4.46 (d, *J* = 5.8 Hz, 2H, C*H*_2_OH), 4.31 (d, *J* = 5.7 Hz, 2H, C*H*_2_OH).

### 3.6. Generation of Intermediate **II** from Reaction of ^Ac^[TM]I + Et_3_N +FF

^Ac^[TM]I (29.9 mg, 0.01 mmoL) and Et_3_N (10.0 mg, 0.01 mmoL) were dissolved in 0.5 mL DMSO-*d*_6_ and transferred into a J. Young-type NMR tube, to which FF (9.6 mg, 0.01 mmoL) in 0.5 mL DMSO-*d*_6_ was added and fully mixed. The mixture was heated to 80 °C on an NMR spectrometer, and the reaction was followed by taking ^1^H NMR spectra of the reaction mixture at predetermined time intervals. After 3 h at 80 °C, the formation of intermediate **II** was indicated by ^1^H NMR spectrum. ^1^H NMR (DMSO-*d*_6_): δ 8.05 (dd, *J* = 1.65, 0.61 Hz, 1H, furan ring proton), 7.45 (dd, *J* = 3.65, 0.61 Hz, 1H, furan ring proton), 6.73 (dd, *J* = 3.65, 1.65 Hz, 1H, furan ring proton), 6.21 (s, 1H, C*H*OH), 3.48 (s, 3H, OC*H_3_*), 2.88 (s, 3H, NC*H_3_*), 2.28 (s, 3H, C=C–C*H_3_*).

## 4. Conclusions

This work has synthesized, and examined catalytic performance of, two new electronically modified thiazolium ILs as inexpensive alternatives to the discrete NHC catalysts for umpolung coupling of bio-derived furaldehyde FF and HMF into chain-extended C_10_ and C_12_ furoins. Comparing to the current thiazolium benchmark catalyst based on ^HO^[TM]Cl, we found that ^AcO^[TM]Cl bearing an electron-donating group at the 5-ring position, when combined with Et_3_N, is the most active and efficient catalyst in this series for coupling of FF, being 4.6 times faster than the current benchmark catalyst system. Thus, the catalyst system with a low catalyst loading of only 0.1 mol % of ^AcO^[TM]Cl and 0.4 mol % Et_3_N, >99% yield of furoin was obtained from the coupling reaction of FF at 60 °C for 1 h. On the other hand, the catalyst system based on ^Ac^[TM]I carrying an electron-withdrawing group at the 5-ring position is the least active and efficient system of the series. For the more challenging coupling reaction of HMF, ^AcO^[TM]Cl and ^HO^[TM]Cl performed equally well, with both achieving 97% yield of DHMF at 120 °C after 3 h, albeit requiring a high catalyst loading of 10 mol %.

The study also yielded the proposed mechanism for the coupling reaction (*c.f.*, Scheme 4). The catalyst responsible for the coupling reaction is the *in situ* generated NHC **I**, upon deprotonation of the thiazolium IL by Et_3_N, which attacks the carbonyl group of FF to form the NHC–FF adduct, intermediate **II**. Upon deprotonation of intermediate **II**, the *in situ* generated enol (**III**), the Breslow intermediate, undergoes nucleophilic attack at the carbonyl group of a second FF molecule to form the second tetrahedral intermediate (**IV**). Collapse of this intermediate, via proton transfer and elimination of **I**, produces the coupling product furoin and regenerates the NHC catalyst, thus closing the catalytic cycle.
